# Femoral fractures in non-accidental trauma and child abuse: biomechanical perspective and insights

**DOI:** 10.3389/fped.2025.1484920

**Published:** 2025-02-25

**Authors:** Giorgio di Laura Frattura, Blaise Cochard, Giacomo De Marco, Oscar Vazquez, Romain Dayer, Dimitri Ceroni

**Affiliations:** ^1^ Pediatric Orthopedics Unit, Pediatric Surgery Service, University Hospitals of Geneva, Genève, Switzerland; ^2^ Division of Orthopedics and Trauma Surgery, Geneva University Hospitals, Geneva, Switzerland

**Keywords:** femoral fracture in childhood, non-accidental injury, biomechanics, child abuse, femoral fracture

## Abstract

Physical abuse remains a global problem that affects children in every country, from every ethnic group, and of all social backgrounds. The fracture of an infant's femur should constitute a red flag to a pediatrician; it must be recognized, recorded, investigated, and potentially result in measures to protect that child. Certain confounding factors, such as the reported mechanism of trauma, could negatively influence the physician's appropriate and unbiased judgment. Indeed, some physically abusive parents may try to explain a femoral fracture as the result of an accidental fall from a changing table or the child's leg getting stuck in bed barriers. This narrative review aimed to provide an overview of this topic and discuss the currently available scientific evidence to better understand the biomechanical mechanisms of femur fractures in infants, thereby definitively putting an end to some popular misconceptions.

## 1 Introduction

Due to its inherent elastic properties and strength, a human femur is very unlikely to break spontaneously without some sort of high-energy trauma. An estimated 4,000 Newtons (N) of force are required to break an adult human femur (1). In infants, femoral fractures should raise a suspicion of abuse if they occur without any significant trauma or underlying metabolic condition, especially if patients are non-ambulatory. Indeed, up to 80% of femoral fractures in non-ambulatory infants are attributable to physical abuse, highlighting the scale of this phenomenon (2). However, it remains extremely difficult to differentiate accidental from non-accidental fractures in infants, mainly because very young children cannot describe the facts surrounding their accident (3). In the emergency room, the infant's parents or relatives will relate their version of the incident. It is unanimously acknowledged, therefore, that abuse should be suspected, or at least considered possible, if care professionals are given an inconsistent or confused version of the accident. One study on this topic showed that 95% of parents reported an “inaccurate or deliberately evasive” story when a femoral fracture was suspected to be associated with non-accidental trauma (2). Thus, it estimated that for every suspected case of abuse reported, 3–10 cases may go unreported and thus undetected (4). Despite various red flags that may suggest a case of abuse, several confounding factors, such as the reported mechanism of trauma, could negatively influence a care professional's appropriate and unbiased judgment (3). Indeed, some parents try to explain away the occurrence of a fracture as an accidental fall from a changing table or the leg getting stuck in the bed's barriers. These two mechanisms are widely reported in emergency rooms despite never having been justified by any scientific evidence or biomechanical analysis (2, 5).

This narrative review's objective was to provide an overview of this topic and discuss the currently available scientific evidence to better understand the biomechanical mechanisms of femur fractures in infants. The review also highlights potential confounding factors related to the specific scenarios often reported by the people in charge of the patient to explain the accident and which might misdirect proper treatment management.

## 2 Epidemiological considerations

Calculating the incidence of pediatric femur fractures due to non-accidental trauma remains a serious challenge for professional caregivers. Femoral fractures should always raise suspicions of abuse, especially among children less than 12 months old, when the trauma's circumstances are unclear, or when there are additional serious injuries, such as bruising, other fractures identified during a skeletal survey, or subdural hemorrhages (6). Different studies have focused on the prevalence rates of non-accidental fractures among infants and young children, ranging from 16% to 79% (posna.org). This variability reflects the heterogeneity of the methods applied to investigating incidences of accidental fracture or abuse. Data come mainly from retrospective studies, some of which were recorded prior to the establishment of programs against child abuse, often before 1990 (7). This point raises the suspicion that abuse may have been under-recognized and under-reported in the past, diminishing the phenomenon's true prevalence. Ongoing prospective studies are trying to elucidate the characteristics (whether anamnestic or biomechanical) of non-accidental trauma among infants and young children.

## 3 Anatomical considerations

The femur is the body's longest and strongest bone. It bows anteriorly, and its diaphysis is a smooth cylinder with differences in cortical thickness all along its length. The *linea aspera* is a major cortical thickening along the femur's posterior aspect to which various groups of muscles from the thigh and the medial and lateral intermuscular septa are attached and also act as a compressive cortical structure themselves (8). It is well known that infants’ bones are weaker and more flexible than those of adults, and the femur is no exception; a child's femur has less compressive strength and stiffness than an adult's (9). After childhood, changes in bone configuration and the ongoing mineralization process during growth explain the major stiffness encountered in adults. Moreover, the geometric cross-section of a child's femoral diaphysis changes drastically during growth, and the cortical shell thickens. Finally, children's bones absorb more energy before breaking than adults’ bones, with energy absorption capacity decreasing approximately threefold between the ages of 3 and 90 due to increased mineralization and a reduced capacity for plastic deformation (10). Consequently, the force required to fracture a 2-year-old's femur is estimated to be twice that needed for a healthy 40-year-old adult's femur. This greater energy absorption is partly due to the higher collagen content and lower mineralization of children's bones, making them more elastic and capable of deforming under stress (11). However, this flexibility should not be mistaken for greater toughness, as toughness—the ability to absorb energy before fracture—is a distinct biomechanical property determined by the total area under the load-deformation curve, encompassing factors beyond elasticity alone. Finally, children's bones, due to their greater elasticity resulting from higher collagen content and lower mineralization, are more flexible and can deform more easily under stress. However, this flexibility should not be mistaken for greater toughness, as the ability to absorb energy before fracture (toughness) is a distinct biomechanical property determined by the total area under the load-deformation curve, which encompasses factors beyond elasticity alone.

Except for distal physeal femur fractures, other femoral fractures’ locations were not thought to be significantly associated with abuse. Corner's fractures have been historically accounted as the most important pattern of abuse for a femoral fracture in children, but nowadays, contradictory data have been encountered in the literature, showing a higher correlation also in diaphyseal fractures (12). This information seems to indicate a greater likelihood of abuse for every type of infant femoral fracture pattern, and these findings should encourage professional caregivers to redefine the characteristics of femoral fractures suggestive of abuse to avoid dangerous and avoidable errors.

## 4 Models of fracture mechanics

Trauma is the most common mechanism resulting in femoral shaft fractures, mostly involving a direct injury or a force transmitted to the femur through the knee or leg. It has been demonstrated that human femurs are not subject to a single loading mode during daily activities but rather to a combination of tensile, compressive, and shear forces (13).

The three mechanisms of trauma that can lead to a childhood femoral fracture are shearing, bending, and torsional stress.

### 4.1 Fractures due to shearing load

These fractures occur when the direction of the deforming force is parallel to the bone's cross-sectional area, applying shearing or tangential stress to the bone. The vector of the force applied to the bone is perpendicular to the bone's axis.

### 4.2 Fractures due to bending load

Bending is a combination of tensile stresses on one side of the bone's neutral axis and compressive stresses on the other. Bending forces typically occur due to a three-point load in which two vectors of force are applied in the same direction at the extremities of the bone, and a third and opposite vector is applied in its middle. The fracture begins on the tension side as bone is weaker in tension than in compression.

### 4.3 Fractures due to torsional load

Torsional load fractures are due to rotational forces. The further the point subject to torque is from its fulcrum, the greater the torsional strength. Such injuries are observed as spiral or oblique fractures. The duration and intensity of the torsional force applied also play determinant roles because bones can endure high loads before failing. A bone that receives a slow load fails twice as fast as one receiving a quick and short load in a brief amount of time.

## 5 Scenario 1: fractures attributed to a fall from the changing table

Most manufacturers of children's dressers and babies’ changing tables agree that their ideal height should be between 73 cm and 80 cm above the floor (14). Falls from changing tables generally occur when children start to roll over by themselves or when the person responsible for them is distracted. It is worth remembering that children generally begin to roll over by themselves at between 6 and 8 months old (15). Rolling to the edge of the table, the child will fall from a height of 80 cm and land flat on either their back, stomach, or side. When falling flat, there is little likelihood that a baby or infant would suffer a femoral fracture. It thus appears that a femoral fracture would require the application of a force against the thigh by an obstacle encountered during the fall. The University of Heidelberg's Institute of Forensic Medicine conducted loading tests that faithfully reproduced a flat fall by a child or infant with landings on the side, the femur or a blunt edge. Nine cadaveric specimens ranging from 2 to 12 months old were subjected to dynamic thigh loads by dropping them horizontally from a height of 70–93 cm onto a horizontal impactor (a blunt edge) that hit the thigh laterally. Speeds at the impact ranged from 13.3 to 15.4 km/h, and the forces registered at the horizontal impactor were from 320 to 600 N. Similarly, dynamic stresses were exerted on the thigh of a deceased 27-month-old infant, generating forces between 1300 and 2370 N. The same authors also tested bending stresses on 18 cadaveric thighs that were loaded using an impactor applied to the middle of the femurs, bending them to the point of fracture. The loads needed to break those bones ranged from 470 N on a 6-day-old baby to 5,700 N on a 15-month-old infant. Considering the forces registered during these tests, the authors concluded that a femoral fracture could not occur if an infant or a child fell from a changing table (5). Moreover, it seems unlikely that a child would fall onto a protruding edge at precisely the point where a shearing load would exert enough stress on the diaphysis to cause a fracture.

## 6 Scenario 2: fractures due to a leg stuck in bed barriers

Populations in Western countries are much more sensitive to the fact that babies sleeping in their parents’ beds have a five times greater risk of succumbing to sudden infant death syndrome (16). Infant beds with barriers have, therefore, naturally become the norm for children from 0 to 3 years old. The space between the bars varies between 4.5 and 6 cm, allowing children to pass a limb between them (17). These beds are potentially dangerous due to the twisting torque that could be exerted should a limb remain stuck between the barriers during sleep. Some parents have thus tried to blame their child's femur fracture on such occurrences. Despite the inconsistencies in these explanations, care professionals still have few scientific arguments with which to challenge them.

Some studies have tried to investigate this by simulating pediatric femoral trauma scenarios using either cadaveric femurs or a computed-tomography-based finite element (18). Conceptually, a lower limb stuck between two bed bars could be subjected to bending and/or torsional mechanical phenomena, with the child applying different intensities of stress while trying to free itself or change position. It is obvious, therefore, that the force generated will be proportional to the child's weight and the forces they can apply. Concerning bending forces, just a few historical studies on cadaveric bones have provided irreplaceable information on babies’, infants’, and very young children's bone strength. Ouyang et al. used a three-point bending test and measured the bending fracture force moments on the femurs of three deceased children aged 2, 2.5, and 3 years old at 29.6, 24.3 and 39.6 N, respectively (19). The femurs of three children aged 1.33, 2, and 2 years old were tested in a similar way by Forman et al., and bending fracture force moments reached 61.4, 61.7, and 65.5 N m (20). The largest study in the literature reporting a three-point bending force test on the femurs of 28 deceased children aged from 1 day to 6 years old was conducted by Miltner and Kallieris in 1989. Quasi-static loading forces were applied to femurs and resulted in bending fracture moments ranging from 7.05 N m (6 days old) to 109.5 N m (6 years old). The forces required to break a femur thus ranged from 500 N (neonates) to 1,350–2,750 N (children 2 years old) (5). One newton is the force needed to accelerate one kilogram of mass at one meter per second squared in the direction of the force applied. On the other hand, the newton-meter is the unit of a torque; one Newton-meter corresponds to the torque resulting from a force of 1 Newton applied perpendicularly to the end of a moment-arm that is one meter long. When compared to moment-arms probably between 5 and 10 cm, it is important to understand that the forces applied will be 10–20 times greater than de moment of force. No studies have directly investigated torsional loads on children's bones to predict failure moments due to this specific type of mechanical stress. Several authors have conducted studies on immature animal femurs to compensate for this lack of data, converting animal age into human age using an equivalence scale such as the study of Bertocci et al., testing torsional forces on immature porcine femurs and registered the failure torque. The porcine equivalents of 1-year-old and 4-year-old human femurs failed at torques of 1.69 N m and 6.77 N m, respectively (21).

Computed-tomography-based finite element models of pediatric femurs have been developed to test bone resistance under bending and torsional forces in infants and very young children. Using 30 femoral models simulating children between 0 and 3 years old, Altai et al*.* demonstrated that “bending to failure” moments ranged from 0.85 to 27.9 N m, with an equivalent load to failure of 97–1,022 N. Moreover, the torque to failure moment was very similar between external and internal rotation, i.e., from 1 to 31.4 N m to 1–30.7 N m, respectively (22).

## 7 Discussion

Most studies focusing on the mechanical properties of immature human and animal femurs have examined static model conditions. We can legitimately consider that static conditions do not accurately represent trauma scenarios. It has been demonstrated that during accidents, human femurs are not subject to a single loading mode but rather a combination of tensile, compressive, and shear forces (8). Evans et al*.* showed that most fractures are associated with a direct impact on the bone (23). Currey et al*.* suggested that a “quasi-static” loading condition, while primarily testing bone strength, may also provide relevant, reliable data that helps to explain the dynamic mechanisms leading to a fracture (11). On the contrary, most other authors disagree, stating that a “quasi-static” testing load probably underestimates the amount of force needed to break a bone subjected to a dynamic impact load (24).

Despite these mechanical studies carried out using either cadaveric human femurs, animal bones, or computed-tomography-based finite element models, it seems unlikely that a newborn or even an infant would be able to generate enough bending or twisting force to break their own femur. Although the results of all these studies do not constitute absolute proof of non-accidental trauma, some of the mechanisms typically used to explain femoral fractures must raise suspicions of physical abuse. An accidental fall from a changing table or a leg getting stuck in a bed barrier have a very low risk of generating a femoral fracture in babies and infants.

In conclusion, these events are highly unlikely and, in our opinion, are suggestive of physical child abuse. However, professional caregivers should always keep in mind that the medical sciences are areas in which statements and terms such as “always”, “never”, and “impossible” should be banned or at least used with great caution.
